# Explicit and implicit attachment and the outcomes of acceptance and commitment therapy and cognitive behavioral therapy for depression

**DOI:** 10.1186/s12888-020-02547-7

**Published:** 2020-04-07

**Authors:** Jacqueline G. L. A-Tjak, Nexhmedin Morina, Wouter J. Boendermaker, Maurice Topper, Paul M. G. Emmelkamp

**Affiliations:** 1 Skils, Dr van Deenweg 98, 8025 BJ Zwolle, the Netherlands; 2 Institute of Psychology, University of Münster, Fliednerstr. 21, 48149 Münster, Germany; 3 Addiction, Development, and Psychopathology (ADAPT) Lab, Department of Psychology, University of Amsterdam, Nieuwe Achtergracht 129 B, 1018 WS Amsterdam, The Netherlands; 4 GGZ-Noord-Holland-Noord, Stationsplein 138, 1703 WC Heerhugowaard, The Netherlands; 5 Department of Clinical Psychology, University of Amsterdam, Nieuwe Achtergracht 129 B, 1018 WS Amsterdam, The Netherlands

**Keywords:** Depression, Treatment, Predictors, Attachment, Implicit, SC-IAT, IAT, Cognitive behavioral therapy, Acceptance and commitment therapy

## Abstract

**Background:**

Attachment theory predicts that patients who are not securely attached may benefit less from psychological treatment. However, evidence on the predictive role of attachment in the effectiveness of treatment for depression is limited.

**Methods:**

Explicit attachment styles, levels of attachment anxiety and attachment avoidance, as well as implicit relational self-esteem and implicit relational anxiety were assessed in 67 patients with major depressive disorder (MDD) receiving Acceptance and Commitment Therapy (ACT) or Cognitive Behavioral Therapy (CBT). ANOVA and hierarchical regression analyses were performed to investigate the predictive power of explicit and implicit attachment measures on treatment outcome.

**Results:**

Explicit attachment avoidance at pre-treatment significantly predicted reduction of depressive symptoms following treatment. Reductions in attachment anxiety and avoidance from pre- to post-treatment predicted better treatment outcomes. Neither one of the implicit measures, nor change in these measures from pre- tot post-treatment significantly predicted treatment outcome.

**Conclusions:**

Our findings show that attachment avoidance as well as reductions in avoidant and anxious attachment predict symptom reduction after psychological treatment for depression. Future research should use larger sample sizes to further examine the role of attachment orientation as moderator and mediator of treatment outcome.

**Trial registration:**

clinicaltrials.gov; NCT01517503.

## Background

Despite a large body of research into the treatment of depression [1], little is known about patient characteristics that can predict response to psychological treatments [2]. Differences in individuals’ interpersonal characteristics may be important in understanding patients’ differential responses to psychological treatment. Attachment theory claims that negative childhood experiences such as growing up with insensible caregivers can lead to mental representations of self as unworthy and of important others as emotionally unavailable. Such representations tend to get in the way of satisfactory interpersonal relationships [3]. Attachment-related anxiety refers to the fear of being abandoned and a desire to be close to partners [4], while attachment-related avoidance relates to down-regulation of emotions by withdrawing from close relationships [5]. Research shows that both adults and adolescents with anxious and avoidant attachment report higher levels of depression than those with secure attachment [6]. Furthermore, empirical data suggest that the patients’ attachment orientation may be an important predictor of treatment outcome. For instance, attachment orientation has been studied as a predictor of the course of symptoms of MDD following hospital discharge [7]. Jones and colleagues [7] found that the combination of high attachment avoidance and low attachment anxiety predicted a lower chance of having MDD 4 months after discharge. Other studies suggest that attachment avoidance, alone or in combination with high attachment anxiety, impacts depression outcome negatively, yet the findings are not entirely consistent [6]. Attachment orientation might further influence the role of the therapeutic alliance as an active treatment ingredient [8, 9]. Interpersonal difficulties may be associated with emotion regulation difficulties and hence have an impact on treatment outcome. Brennan and colleagues [10] conceive individual differences in attachment in terms of anxiety and avoidance as underlying dimensions of emotional and behavioral regulation.

Attachment is often measured with self-report measures, like the Experiences in Close Relationships (ECR) [10]. Evidence from self-report studies supports the theoretical assumptions about attachment security and attachment anxiety. However, findings are less coherent on the relationship between attachment and psychotherapy outcome [6]. This is perhaps related to the use of self-report measures, which may have several drawbacks [11]. Automatic processes are assumed to play a crucial role in attachment behavior [5], and research on the relationship between attachment and psychotherapy outcome may profit from the inclusion of implicit assessment of attachment. Implicit measures, such as the Implicit Association Test (IAT) [12] are used to map automatic processes that are more difficult to measure with explicit measurements because they are unconscious or because they are influenced by social evaluations [13]. Implicit measures are known to be capable to predict behavior [14, 15]. The IAT in particular has been shown to predict spontaneous behavior [11]. Dewitte and colleagues [16] used the IAT to assess the relationship of relational self-esteem and relational anxiety with attachment style. The authors concluded that there was a significant relationship between the degree of implicit relational self-esteem and implicit relational anxiety, as measured by the IAT, and individual differences in attachment style.

An IAT consists of several blocks in which participants are instructed to categorize words as quickly as possible into different categories by pressing two separate response-buttons. Participants discriminate target items consisting of certain concepts and their opposites, for instance ‘me’ and ‘not-me’. In addition, they sort attribute-items into two categories, like relationally worthy and relationally worthless. Attributes and targets are combined in several ways. The hypothesis is that association strengths between attribute-items, target items and categories influence performance. The IAT uses complementary pairs of concepts and attributes, which limits it use to measuring the relative strengths of pairs of associations rather than absolute strengths of single associations. In the context of measuring self-concepts the choice of a complement is not always obvious. For instance, ‘not me’ or ‘other’ is far less specific than ‘I’ or ‘me’. Karpinski and Steinman [17] designed the Single Category IAT (SC-IAT) as a modification of the IAT procedure to measure the evaluative associations with a single category or attitude object. This resolves the problems of the IAT mentioned above, while keeping the advantages, as the SC-IAT shares many properties with the IAT.

The aim of this study was to investigate the effects of initial levels of attachment, and changes in attachment during treatment on the level of symptoms and quality of life in the treatment of depression. Attachment was measured both explicitly and implicitly. We used the ECR to measure attachment explicitly, specifically the two subscales of attachment avoidance and attachment anxiety. To measure attachment implicitly we based ourselves on available measures, from the work of Dewitte and colleagues [16]. For attachment anxiety we used their measure to create a SC-IAT. To keep the link to their work clear, we have called this measure ‘relational anxiety’. Unfortunately, the authors did not develop a measure for attachment avoidance. This means that we could not test attachment avoidance both explicitly and implicitly. We decided to use the measure for relational self-esteem from Dewitte and colleagues [16] to create a second SC-IAT measure related to attachment. This measure is called ‘relational self-esteem’. Based on the attachment theory [6], for the explicit measures we expected 1) both high initial levels of attachment anxiety and high initial levels of attachment avoidance to predict less response to treatment and 2) an interaction effect in which the combination of low attachment anxiety and low attachment avoidance predicts a superior response to treatment as compared to all other combinations of attachment anxiety and avoidance. Furthermore, based on earlier research [18, 19] we expected that 3) attachment anxiety and attachment avoidance scores would decrease from pre-treatment to post-treatment and that this change would predict a beneficial treatment outcome. On our implicit measures, based on the work of Dewitte and colleagues [16], we expected that 4) lower pre-treatment implicit relational self-esteem scores and higher pre-treatment implicit relational anxiety scores would be associated with less beneficial treatment outcomes. In accordance with our expectations concerning our explicit measures, we expected that 5) implicit relational anxiety scores would decrease from pre-treatment to post-treatment whereas implicit relational self-esteem scores would increase. Furthermore, we expected that this change would predict a beneficial treatment outcome.

## Methods

### Participants

We recruited study participants in specialized out-patient care clinic in the Netherlands. Eligible patients had been referred for treatment by their general practitioner or other mental health practitioners. Patients were between 18 and 65 years and were diagnosed with MDD as the principal diagnosis, as assessed with the Structured Clinical Interview for DSM-IV Axis I Disorders (SCID) [20]. Patients with comorbid disorders were excluded only if the comorbid disorder consisted of: bipolar, psychotic, borderline or anti-social personality, substance dependence, or organic brain syndrome. Antidepressant medication was required to be on a stable dose for at least 2 weeks. Of the 601 assessed for eligibility, 99 were randomized and 82 received treatment within our project. At post-treatment, 67 subjects filled in all measures. A detailed description of the method, treatment and the results on outcome can be found elsewhere [21].

### Measurements

#### Outcome measures

We applied the Quick Inventory of Depressive Symptomatology (QIDS) [22] to measure depressive symptoms. This self-report contains 16-items and assesses several domains of depression, including sleep disturbance, psychomotor activity and changes in appetite or weight. The QIDS total score ranges from 0 to 27. A score of 0–5 is considered to be within the normal range. We assessed the QIDS at pre- and post-treatment. Cronbach’s αs were .68 pre-treatment and .85 post-treatment.

We used the European Health Interview Surveys Quality of Life Scale (EUROHIS) [23] to assess quality of life. It consists of 8 items (overall quality of life, general health, energy, daily life activities, esteem, relationships, finances, and home). Total scores can range from 8 to 40, with higher scores indicating higher quality of life. The EUROHIS showed acceptable cross-cultural performance and a satisfactory discriminant validity [24]. We assessed the EUROHIS at pre- and post-treatment. Cronbach’s αs were .73 pre-treatment and .83 post-treatment.

#### Potential predictors of treatment outcome

To measure explicit attachment aspects, we used the Dutch version [25] of the Experience in Close Relationships [10]. We used the two subscales, which measure the dimensions attachment anxiety and attachment avoidance. The questionnaire consists of 36 items. The Dutch version was found to be valid and reliable in a general sample of the Dutch population [25], with Cronbach’s α = .88 for the avoidance subscale and Cronbach’s α = .86 for the anxiety subscale. We assessed the ECR at pre- and post-treatment. In our study, Cronbach’s αs were .90 and .88 for attachment anxiety pre- and post-treatment and .91 and .93 for attachment avoidance pre- and post-treatment.

To measure implicit aspects of attachment, we used SC-IATs [17]. The two types of SC-IATs used in this study related to relational self-esteem and relational anxiety were based on Dewitte and colleagues [16]. Prior to each SC-IAT, participants were instructed to imagine that an important attachment figure would go away for a longer period of time. The aim of this priming assignment was to activate the participant’s attachment system [16]. Each SC-IAT consisted of two critical test blocks that were preceded by a practice block. In each upper corner of the screen there were two fixed attribute categories for each SC-IAT, either ‘loved’ and ‘unwanted’ or ‘calm’ and ‘anxious’. In the two trial blocks a target label was placed under one of the attribute categories. The target label was ‘me’ for both SC-IATs. Both the sequence of the SC-IATs and the place where the target label first appeared were counterbalanced, to counter sequence effects [26]. Participants had to place words that appeared on the screen as quickly as possible under the right attribute category with any target label. The premise was that the sorting becomes easier (i.e., relatively shorter reaction times) when a target and attribute that share the same response key are strongly associated than when they are weakly associated. To determine which association was stronger, the average reaction times of block 2 and block 3 were compared per SC-IAT. A faster average reaction time was, as with the IAT, interpreted as a stronger association in the memory between the attribute category and the target label in question [27]. The reliability of the SC-IAT is comparable to the usual reliability of IAT measurements [17]. We assessed the SC-IATs before and after treatment.

### Treatment

Participants were randomly assigned to either Cognitive Behavioral Therapy (*n* = 38) or Acceptance and Commitment Therapy (*n* = 44). Treatment in both conditions consisted of a minimum of 8 and a maximum of 20 sessions over 30 weeks. Sessions generally lasted 45–55 min. CBT was provided based on a manual that is consistent with standard CBT for depression [28] and included both behavioral and cognitive aspects in an integrated fashion [29]. ACT was provided through the treatment manual for depression developed for this study by A-Tjak [30], based on Hayes, Strosahl, and Wilson [31], Zettle [32], and Robinson and Strosahl [33]. The manual consists of 16 sessions addressing acceptance, defusion, an observing perspective and present moment awareness in the first eight sessions. The subsequent part of the treatment addressed behavioral change through values clarification and shaping committed action.

### Procedure

The data used in the present study are part of a randomized controlled effectiveness trial comparing CBT and ACT [21]. In short, we found that at post-treatment, remission rates from depression were 75 and 80% for the ACT and CBT condition, respectively. Patients in both conditions further reported significant and large reductions of depressive symptoms and improvement on quality of life from pre- to post-treatment as well as at 6-month follow-up. Our findings indicated no significant differences between the two intervention groups at post-treatment and at 6-month follow-up. For the present study, we combined the data of patients of both intervention groups. Data were collected at pre-treatment and following treatment at the treatment site. Participants first answered questions about demographics and filled in questionnaires. After filling in questionnaires at the computer, they completed the SC-IAT.

#### Data reduction SC-IATs

Processing of the SC-IAT data took place on the basis of improved scoring algorithm [34]. Incorrectly answered trials were repeated. Responses to the second trial were removed. In accordance with the scoring algorithm, all trials with latencies in reaction time over 10 s were removed. When participants had a reaction time shorter than 300 ms on more than 10% of the trials, all trials of these participants were also removed. The average reaction time per participant was calculated for block 2 and block 3. The trials in which the participant gave the wrong answer were replaced by the block average plus twice the standard deviation of the block in question. Subsequently, the new block means were calculated with the corrected data and associated standard deviations. Finally, the bias score per SC-IAT was calculated by dividing the block average of block 3 minus the block average of block 2 by the standard deviation. As such, a positive bias score indicates, per SC-IAT, a positive self-esteem, and a high relational anxiety, respectively.

#### Statistical analyses

The data were screened for outliers and normality. All variables were found to be normally distributed. Incomplete data sets were not used. Regression analyses and repeated measures ANOVAs were performed with SPSS version 25. Datasets of dropouts, when post-treatment data were available (*n* = 11), were used.

## Results

Table 1 shows means and standard deviations of all measured variables. We calculated correlations between the implicit attachment measures and the two subscales of the ECR and found no significant correlations (see Table 2).

**Table 1:** Means and standard deviations of depression, quality of life, explicit and implicit attachment

Measure	Mean pre-treatment	Standard deviation pre-treatment	Mean post-treatment	Standard deviation post-treatment
Explicit	(*n =* 82)		(*n =* 67)	
QIDS	14.79	4.32	7.09	6.01
EUROHIS	20.45	4.66	25.64	5.53
ECR-anxiety	69.60	19.72	63.55	16.47
ECR-avoidance	58.88	18.75	54.16	17.67
Implicit	(*n =* 78)		(*n =* 59)	
SC-IAT-RSE	−.16	.79	−.23	.31
SC-IAT-RA	.19	.35	.29	.36

Note: *QIDS* Quick Inventory for Depressive Symptomatology, *EUROHIS* European Health Interview Surveys Quality of Life Scale, *ECR* Experiences in Close Relationships Questionnaire, *SC-IAT* Single Category Implicit Association Test, *RSE* relational self-esteem, *RA* relational anxiety

**Table 2:** Correlations between all measures (with number of participants between brackets)

	2	3	4	5	6	7	8	9	10	11	12
1. QIDS pre	.34**	−.44**	−.36**	.19	.18*	.10	.21*	−.02	.06	−.00	−.07
	(67)	(82)	(67)	(82)	(67)	(82)	(67)	(77)	(64)	(77)	(64)
2. QIDS post		−.20*	−.54**	.11	.33**	−.26**	.01	−.13	.03	.08	−.02
		(67)	(67)	(67)	(67)	(67)	(67)	(64)	(64)	(65)	(64)
3. EUROHIS pre			.37**	−.15	−.15	−.20*	−.10	−.07	−.01	.08	.01
			(67)	(82)	(67)	(82)	(67)	(77)	(64)	(78)	(64)
4. EUROHIS post				−.08	−.34**	.06	−.23**	.10	.04	−.09	.05
				(67)	(67)	(67)	(67)	(64)	(64)	(65)	(64)
5. ECR-anxiety pre					.50**	.06	.02	.09	.04	−.01	.01
					(67)	(82)	(67)	(77)	(64)	(78)	(64)
6. ECR-anxiety post						−.16	−.01	.03	−.04	.07	.04
						(67)	(67)	(64)	(64)	(65)	(64)
7. ECR-avoidance pre							.53**	.08	−.14	−.09	.09
							(67)	(77)	(64)	(78)	(64)
8. ECR-avoidance post								−.04	−.06	.01	−.02
								(64)	(64)	(65)	(64)
9. SC-IAT-RSE pre									.03	−.22	.05
									(62)	(77)	(62)
10. SC-IAT-RSE post										.01	−.40**
										(62)	(64)
11. SC-IAT-RA pre											−.01
											(62)
12. SC-IAT-RA post											

*Note: QIDS* Quick Inventory for Depressive Symptomatology, *EUROHIS* European Health Interview Surveys Quality of Life Scale, *ECR* Experiences in Close Relationships Questionnaire, *pre* pre-treatment, *post* post-treatment, *SC-IAT* single category Implicit Associations Test, *RSE* relational self-esteem, *RA* relational anxiety. * p < .05, ** p < .01

### The predictive value of attachment anxiety, attachment avoidance and their interaction on the outcome measures (hypotheses 1 and 2)

A series of hierarchical multiple regression analyses were conducted with the QIDS and EUROHIS as the outcome measure and the ECR anxiety and avoidance subscales and the interaction term of those subscales as predictors. Predictors were centered to avoid multicollinearity. To control for initial levels of depressive symptoms and quality of life, the pre-treatment scores of the outcome measure (i.e., QIDS or EUROHIS) were entered in step 1, the predictor variables were entered in step 2 and the interaction term in step 3. Table 3 shows the results of the regression analyses. Our first hypothesis could not be confirmed. Attachment anxiety did not predict treatment outcome, neither for depressive symptoms, nor for quality of life. Also, attachment avoidance did not predict quality of life outcome. The only significant finding, was the prediction of attachment avoidance on the post-treatment QIDS scores (*p* = .001), over and above the pre-treatment QIDS scores, with ΔR^2^ = .15, *p* = .001. Contrary to our first hypothesis, the value of the unstandardized beta indicates that higher pre-treatment avoidance scores on the ECR were associated with lower symptom levels of depression at post-treatment. Our second hypothesis could not be confirmed, we found no interaction effect for attachment anxiety and attachment avoidance predicting treatment outcome, neither for depressive symptoms, nor for quality of life.

**Table 3:** Hierarchical regression analysis: explicit attachment dimensions predicting symptom levels of depression and quality of life

Measure	B	SE B	β	*p*	sr^2^
*a) ECR-subscales centered predicting QIDS at post-treatment*					
Step 1					
Constant	−.89	2.41		.71	
QIDS pre-treatment	.56	.16	.39	**.001**	.39
Step 2					
Constant	−2.24	2.32		.34	
QIDS pre-treatment	.64	.16	.45	**<.001**	.44
ECR-anxiety	−.02	.04	−.05	.67	−.05
ECR-avoidance	.13	.04	.39	**.001**	.38
Step 3					
Constant	−2.16	2.35		.36	
QIDS pre-treatment	.64	.16	.45	**<.001**	.43
ECR-anxiety	−.02	.04	−.06	.61	−.05
ECR-avoidance	.13	.04	.39	**.001**	.37
Interaction between ECR-anxiety and ECR-avoidance	.00	.00	.04	.73	.04
b) *ECR-subscales centered predicting EUROHIS at post-treatment*					
Step 1					
Constant	12.89	2.75		**<.001**	
EUROHIS pre-treatment	.61	.13	.51	**<.001**	.51
Step 2					
Constant	12.46	2.82		**<.001**	
EUROHIS pre-treatment	.63	.13	.53	**<.001**	.51
ECR-anxiety	.01	.03	.03	.76	.03
ECR-avoidance	−.04	.03	−.13	.23	−.13
Step 3					
Constant	12.44	2.84		**<.001**	
EUROHIS pre-treatment	.63	.13	.53	**<.001**	.51
ECR-anxiety	.01	.03	.02	.86	.02
ECR-avoidance	−.04	.03	−.14	.22	−.14
Interaction between ECR-anxiety and ECR-avoidance	.00	.00	.05	.65	.05

Note: a) R^2^ = .16 for step 1 (*p* = .001); ΔR^2^ = .15 for step 2 (*p* = 0.02); ΔR^2^ = .00 for step 3 (*p* = 0.73); b) R^2^ = .26 for step 1 (*p* < 0.001; ΔR^2^ = .02 for step 2 (*p* = .48); ΔR^2^ = .00 for step 3 (*p* = .65). *QIDS* Quick Inventory for Depressive Symptomatology, *EUROHIS* European Health Interview Surveys Quality of Life Scale, *ECR* Experiences in Close Relationships Questionnaire. Significant *p*-values (*p* < .05) are marked in bold

### The predictive value of changes in attachment anxiety and attachment avoidance on the outcome measures (hypothesis 3)

In support of hypothesis 3, a series of repeated measures ANOVAs demonstrated a significant pre- to post-treatment decrease of attachment anxiety, ***F*(1, 66) = 5.94,*p*** **= .02,***η*_p_^2^ = .08, as well as a significant pre- to post-treatment decrease of attachment avoidance, ***F*(1, 66) = 4.33,*p*** **= .04,***η*_p_^2^ = .06. A series of hierarchical multiple regression analyses were conducted with the pre- to post-treatment change in ECR anxiety and avoidance scales as predictors and the QIDS and EUROHIS as the outcome measures. As can be seen in Table 4, pre-treatment scores of the outcome measure were entered in step 1 to control for initial levels of depressive symptoms and quality of life, and the predictor variables were entered in step 2. In line with our third hypothesis, pre- to post-treatment decreases significantly predicted lower depression scores at post-treatment, ΔR^2^ = .12, *p* = .002 for attachment anxiety, and ΔR^2^ = .18, *p* < .001 for attachment avoidance. Similarly, pre- to post-treatment decreases in attachment anxiety and attachment avoidance predicted higher quality of life post-treatment, ΔR^2^ = .12, *p* = .001 for attachment anxiety, and ΔR^2^ = .19, *p* < .001 for attachment avoidance.

**Table 4:** Hierarchical regression analysis: changes in attachment dimensions predicting levels of depression and quality of life

Measure	B	SE B	β	*p*	sr^2^
*a) ECR-anxiety T2-T1 predicting QIDS at post-treatment*					
Step 1					
Constant	−.89	2.41		.71	
QIDS pre-treatment	.56	.16	.39	**.001**	.39
Step 2					
Constant	.58	2.30		.80	
QIDS pre-treatment	.50	.15	.35	**.002**	.35
ECR-anxiety T2-T1	.15	.05	.35	**.002**	.35
*b) ECR-anxiety T2-T1 predicting EUROHIS at post-treatment*					
Step 1					
Constant	12.89	2.75		**<.001**	
EUROHIS pre-treatment	.61	.13	.51	**<.001**	.51
Step 2					
Constant	13.65	2.55		**<.001**	
EUROHIS pre-treatment	.54	.12	.45	**<.001**	.45
ECR-anxiety T2-T1	−.14	.04	−.35	**.001**	−.35
*c) ECR-avoidance T2-T1 predicting QIDS at post-treatment*					
Step 1					
Constant	−.89	2.41		.71	
QIDS pre-treatment	.56	.16	.39	**.001**	.39
Step 2					
Constant	.88	2.19		.69	
QIDS pre-treatment	.48	.15	.34	**.002**	.34
ECR-avoidance T2-T1	.18	.04	.43	**<.001**	.43
*d) ECR-avoidance T2-T1 predicting EUROHIS at post-treatment*					
Step 1					
Constant	12.89	2.75		**<.001**	
EUROHIS pre-treatment	.61	.13	.51	**<.001**	.51
Step 2					
Constant	12.00	2.41		**<.001**	
EUROHIS pre-treatment	.62	.11	.52	**<.001**	.52
ECR-avoidance T2-T1	−.17	.04	−.43	**<.001**	−.43

Note: a) R^2^ = .16 for step 1; ΔR^2^ = .12 for step 2; b) R^2^ = .26 for step 1; ΔR^2^ = .12 for step 2; c) R^2^ = .16 for step 1; ΔR^2^ = .18 for step 2; d) R^2^ = .26 for step 1; ΔR^2^ = .19 for step 2. *QIDS* Quick Inventory for Depressive Symptomatology, *EUROHIS* European Health Interview Surveys Quality of Life Scale, *ECR* Experiences in Close Relationships Questionnaire, *T1* pre-treatment, *T2* post-treatment, T2-T1 is the difference in scores from pre- to post-treatment, Significant *p*-values (*p* < .05) are marked in bold

### The predictive value of implicit relational anxiety and relational self-esteem on the outcome measures (hypothesis 4)

A series of hierarchical multiple regression analyses were conducted to examine the degree to which the SC-IAT attachment measures at pre-treatment predicted treatment outcome as assessed with the QIDS and EUROHIS. Here too, pre-treatment scores of the QIDS and EUROHIS were entered in step 1 to control for initial levels of depressive symptoms and quality of life (see Table 5). Our expectations were not confirmed, as none of the implicit attachment scores provided significant explanatory power to the post-treatment scores on the QIDS and EUROHIS. This means that neither implicit relational anxiety nor relational self-esteem predicted treatment response.

**Table 5:** Hierarchical regression analysis: implicit attachment predicting symptom levels of depression and quality of life

Measure	B	SE B	β	*p*	sr^2^
*a) SC-IAT-RA predicting QIDS at post-treatment*					
Step 1					
Constant	−1.53	2.47		.54	
QIDS pre-treatment	.61	.17	.42	**.001**	.42
Step 2					
Constant	−1.57	2.49		.53	
QIDS pre-treatment	.60	.17	.41	**.001**	.41
SC-IAT-RA	1.01	2.02	.06	.62	.06
*b) SC-IAT-RA predicting EUROHIS at post-treatment*					
Step 1					
Constant	12.94	2.80		**<.001**	
EUROHIS pre-treatment	.60	.13	.51	**<.001**	.51
Step 2					
Constant	13.94	2.83		**<.001**	
EUROHIS pre-treatment	.60	.13	.50	**<.001**	.50
SC-IAT-RA	−2.07	1.73	−.13	.24	−.13
*c) SC-IAT-RSE predicting QIDS at post-treatment*					
Step 1					
Constant	−2.26	2.45		.36	
QIDS pre-treatment	.65	.17	.45	**<.001**	.45
Step 2					
Constant	−2.94	2.50		.24	
QIDS pre-treatment	.66	.17	.45	**<.001**	.45
SC-IAT-RSE	−2.48	1.96	−.14	.21	−.14
*d) SC-IAT-RSE predicting EUROHIS at post-treatment*					
Step 1					
Constant	11.39	2.73		**<.001**	
EUROHIS pre-treatment	.69	.13	.56	**<.001**	.56
Step 2					
Constant	11.54	2.70		**<.001**	
EUROHIS pre-treatment	.71	.13	.58	**<.001**	.58
SC-IAT-RSE	2.61	1.66	.16	.12	.16

Note: a) R^2^ = .20 for step 1; ΔR^2^ = .02 for step 2; b) R^2^ = .17 for step 1; ΔR^2^ = .00 for step 2; c) R^2^ = .32 for step 1; ΔR^2^ = .03 for step 2; d) R^2^ = .26 for step 1; ΔR^2^ = .03 for step 2. *SC-IAT* single category Implicit Associations Test, *RSE* relational self-esteem, *RA* relational anxiety, *QIDS* Quick Inventory for Depressive Symptomatology, *EUROHIS* European Health Interview Surveys Quality of Life Scale. Significant p-values (*p* < .05) are marked in bold

### The predictive value of changes in implicit relational anxiety and relational self-esteem on the outcome measures (hypothesis 5)

In contrast to the hypotheses, a series of repeated measures ANOVAs demonstrated no significant pre- to post-treatment decreases in implicit relational self-esteem, ***F*(1, 61) = .12,*p*** **= .74,***η*_p_^2^ = .00, as well as no significant pre- to post-treatment changes in implicit relational anxiety, ***F*(1, 61) = 1.94,*p*** **= .17,***η*_p_^2^ = .03. Therefore, we did not pursue with the planned regression analyses.

## Discussion

The aim of this study was to investigate the effects of 1) initial levels of attachment, and 2) changes in attachment during treatment on the level of symptoms and quality of life in the treatment of depression. Attachment was measured both explicitly with self-report measures and implicitly with SC-IATs. Our first hypothesis was that both high initial levels of attachment anxiety and attachment avoidance, measured explicitly, would predict less response to treatment. Contrary to our expectations, neither attachment anxiety nor attachment avoidance predicted better quality of life after treatment. Surprisingly, higher attachment avoidance, but not higher attachment anxiety, as measured with the ECR, predicted more symptom reduction. Our second hypothesis was that the combination of low attachment anxiety and low attachment avoidance would predict good response to treatment and all other combinations would predict less response to treatment. Yet, we found no significant interaction effects. Our third hypothesis was that attachment anxiety and attachment avoidance scores would decrease from pre-treatment to post-treatment and that this change would predict treatment outcome. This hypothesis was partially met. Both attachment anxiety and attachment avoidance scores decreased from pre-treatment to post-treatment. We found a relationship between this decrease and the outcome for depression symptoms, but not for quality of life. Our fourth hypothesis was that lower pre-treatment implicit relational self-esteem scores and higher pre-treatment implicit relational anxiety scores would be associated with less treatment outcomes. Contrary to our expectations, we found no significant predictive relationship between implicit attachment measures and treatment outcome. Finally, in contrast to our fifth hypothesis, we found no indication that relational self-esteem or relational anxiety scores changed from pre-treatment to post-treatment ruling out the possibility that these changes predicted treatment outcome.

Attachment anxiety, measured explicitly, and relational anxiety, measured implicitly, assessed at pre-treatment were not a significant predictor of either depressive symptoms or quality of life at post-treatment. One reason for this finding could be that people who are anxiously attached, seek support from people they feel close to. Depressive symptoms may not be related to interpersonal problems for those who are anxiously attached and in a secure relationship. Attachment anxiety may be more pronounced when the relationship is threatened or absent. However, we were not able to assesses to what extent this applied to our sample, as we had not measured the quality of the relationship. The number of participants in the current study may not have been large enough to detect potential associations between attachment and depression or quality of life. However, other studies have produced similar results with regard to explicitly measured attachment anxiety. In the study by Woods and colleagues [35] attachment anxiety did not predict change in depression. McBride and colleagues [18] also found that attachment anxiety was not related to treatment outcome. Then again, results from previous research are mixed [6]. Perhaps a third variable, like the quality of interpersonal relationships, influences the relationship between attachment anxiety and depression or quality of life. Accordingly, research with larger samples is needed to further investigate this relationship.

At first sight, it seems rather surprising that while higher scores of attachment avoidance at pre-treatment were related to better depression outcomes, the decrease in both subscales of the ECR were related to a reduction in depressive symptoms. It is possible that attachment avoidance is a moderated mediator. This would mean that especially people high in attachment avoidance profit from psychological therapy, or at least, from cognitive behavioral therapy approaches. McBride and colleagues [18] found that high attachment avoidance was associated with greater reductions in depressive symptoms following CBT as compared to Interpersonal Therapy (IPT). The authors suggested that this finding might be explained by the explicit emphasis on improving individuals’ relationships and interpersonal interactions in IPT rather than CBT. Working on the relationship could be too threatening for people who regulate their emotions by avoiding closeness. Ravitz and colleagues [36] suggest from previous research that attachment avoidance may interfere with treatment response in IPT, because it addresses interpersonal functioning. Woods and colleagues [35] found in a sample of women dissatisfied with their romantic relationship that more avoidant women experienced significantly better treatment outcomes. An explanation could be that avoidance prevented engagement in negative interactions with their partner, keeping dissatisfaction low. Additionally, these researchers found that symptoms worsened when they perceived their partners as overinvolved. Perhaps being in therapy lessens the involvement of partners, who expect therapists to help the patient, which in turn leads to improvement in symptoms. Attachment avoidance may also influence non-romantic relationships. Hardy and Barkham [9] found that attachment avoidance correlated with difficulties with work colleagues, with relationships at home and in social life.

Time may be another important factor to understand the differences in outcome regarding attachment avoidance. Perhaps in the short term, treatments that do not directly focus on interpersonal effectiveness lead to better depression outcome. In the long term, helping people to connect and improve relationships may be more challenging, but also improving quality of life and perhaps protect from relapse into depression. According to Zilcha-Mano and colleagues [37], this could be done by encouraging feelings of intimacy in the patient-therapist relationship, which is not customary in CBT. While attachment avoidance was related to depressive symptoms in our study, it was not related to quality of life. This indicates that symptom reduction and improvement of quality of life are differently influenced by patient characteristics like attachment avoidance. Attachment orientation may not be related to quality of life during treatment. Perhaps changes in attachment orientation need more time before their influence on quality of life becomes measurable. Future research should focus on comparing therapies for depression, that do not focus explicitly on interpersonal relationships with therapies that do, related to attachment avoidance and relationship satisfaction in the long run.

We found no interaction effect in our study, possibly due to lack of power. Another possibility is that the ECR may not be suited to adequately assess the interaction between attachment dimensions. Jones and colleagues [7] found the combination of high attachment avoidance with low attachment anxiety to be predictive of a lower chance of having MDD at 4-months after discharge. The combination of high attachment avoidance and high attachment anxiety was predictive of a higher risk of having MDD at 4-months after discharge, at a trend level. Jones and colleagues did not perform an analysis with an interaction term, but used instead the combination of attachment anxiety and avoidance with the Relationship Questionnaire (RQ). Constantino and colleagues [19] found that adults who were low on both anxiety and avoidance were more likely to remit from depression, using the Relationship Scales Questionnaire. Cyranowski and colleagues [38] found that securely attached women as assessed with the RQ, responded faster than women who were high on both attachment anxiety and avoidance. To the best of our knowledge, our study is the first to use a SC-IAT to measure relationship self-esteem and relational anxiety.

We found no significant relationship between treatment outcome and both implicit measures, or the change in those measures. Neither did we find a significant correlation between the implicit measures and the ECR subscales (see Table 2). With regard to relational self-esteem, this may come as no surprise as the SC-IAT measured a different aspect of attachment than the ECR. However, we also found no correlation between the subscale of attachment anxiety with relational anxiety as measured with the SC-IAT. This is in line with the findings of Venta and colleagues [39] who found no relationship between attachment IATs and romantic partner attachment as measured with the ECR revised. The authors report that their finding is representative, to some degree, to all other attachment IAT studies. Furthermore, according to the literature on Emotion Response Coherence, it turns out that different measures of emotional responses are often not associated [40, 41]. To make sense of the data, Evers and colleagues [40] propose a dual-process framework consisting of two largely independent systems: an automatic (implicit) and a reflective (explicit) system. Implicit and explicit motivational systems are also viewed as distinct [42]. This underscores the importance of applying both explicit and implicit measures when investigating human behavior. However, in our study, we did not directly compare explicit and implicit measures. Furthermore, it remains unclear whether an explicit focus during treatment on implicit cognitions might have led to change in implicit cognitions, which could have influenced treatment outcome. According to Beevers [43] effortful explicit processing could override implicit negative responses. There is some evidence suggesting that an individual’s awareness of implicit responses can increase the efficacy of CBT [44].

Dewitte and colleagues [16] found levels of implicit relational self-esteem and implicit relational anxiety as measured by the IAT to be related to the attachment dimensions. There were several differences between their study and ours, that may have influenced the results. An important difference is that their study was not conducted within a clinical sample. In addition, we did not specify in our study on what attachment figure patients had to focus on before taking the SC-IAT assessment. Research by Fraley and colleagues [45] suggests that the context of the attachment figure is relevant. Perhaps specifying the attachment figure, as was done by Dewitte and colleagues [16], would have primed implicit attachment aspects better. Another difference is that they used the revised version of the ECR, whereas we used the original ECR [10]. More importantly, the SC-IATs as applied in our study may lack construct validity, in relation to psychopathology in general and depression specifically. Associations between single words may not evoke the same meaning as full sentences such as used in questionnaires. Creating implicit measures measuring specific content may need considerable fine-tuning. Perhaps using double categories, such as used in the IAT is necessary to evoke the right purport. Hofmann and colleagues [15] came to the conclusion that an increasing conceptual correspondence between measures leads to increased correlations. They also found that correlations can be enhanced through spontaneity of self-reports. Another possibility for our null finding is that there is no relationship between implicit attachment as measured with the SC-IAT or IAT and treatment outcome. Measuring attachment by means of implicit measures is still in its infancy. Future research should compare the IAT and SC-IAT directly, make sure participants are adequately primed and construct SC-IATs with content based on explicit measures.

A considerable strength of our study into potential implicit predictors of outcome in depression treatment is that we used a clinical sample. Many studies on implicit measures have been conducted with academic samples, which may lack generalizability to clinical populations [44]. Furthermore, we applied both explicit and implicit measures. However, our study has also some limitations. The lack of results regarding the impact of attachment anxiety, measured implicitly and explicitly, implicit relational self-esteem, and the interaction effect of the two subscales of the ECR may be due to insufficient power of our study. The relative low number of participants per treatment model (CBT and ACT) prohibited a comparison of the predictors per treatment condition. Yet, treatment conditions did not significantly differ in treatment outcome [21]. Additionally, no manipulation checks were performed to control whether instructions to imagine that an important attachment figure would go away for a longer period of time were successful. Nor did we specify what kind of relationship there had to be with the attachment figure. Furthermore, we did not assess whether participants had been romantically involved.

## Conclusion

To conclude, our study suggests that attachment orientation is a relevant predictor of treatment outcome for depression. Future research is needed to investigate the validity of current and prospective measurements of implicit attachment. Furthermore, clinical trials with larger samples and multiple data points are needed to gain better insight into how attachment may serve as a moderator and mediator of treatment outcome.

## Data Availability

The datasets used and/or analyzed during the current study are available from the first author on reasonable request.

## Abbreviations

ACT: Acceptance and Commitment Therapy
CBT: Cognitive Behavioral Therapy
ECR: Experiences in Close Relationships
EUROHIS: European Health Interview Surveys Quality of Life Scale
IAT: Implicit Association Test
IPT: Interpersonal Therapy
MDD: Major Depressive Disorder
QIDS: Quick Inventory of Depressive Symptomatology
QIDS-SR: Quick Inventory of Depressive Symptomatology Self-Report
SC-IAT: Single Category Implicit Association Test(s)
SCID I: Structured Clinical Interview for DSM-IV Axis I Disorders
